# Dietary fatty acid composition alters 11β-hydroxysteroid dehydrogenase type 1 gene expression in rat retroperitoneal white adipose tissue

**DOI:** 10.1186/1476-511X-9-111

**Published:** 2010-10-08

**Authors:** Sakamuri SS Vara Prasad, Shanmugam S Jeya Kumar, Putcha Uday Kumar, Syed SYH Qadri, Ayyalasomayajula Vajreswari

**Affiliations:** 1 Department of Biochemistry, National Institute of Nutrition, Indian Council of Medical Research, Jamai Osmania PO, Hyderabad-500 604, Andhra Pradesh, India; 2 Department of pathology, National Institute of Nutrition, Indian Council of Medical Research, Jamai Osmania PO, Hyderabad-500 604, Andhra Pradesh, India

## Abstract

The enzyme 11β-hydroxysteroid dehydrogenase type 1 (11β-HSD1) amplifies intracellular glucocorticoid action by converting inactive glucocorticoids to their active forms *in vivo*. Adipose-specific overexpression of 11β-HSD1 induces metabolic syndrome in mice, whereas 11β-HSD1 null mice are resistant to it. Dietary trans and saturated fatty acids (TFAs and SFAs) are involved in the development of metabolic syndrome, whereas polyunsaturated fatty acids (PUFA) offer protection against this. Here, we report the effects of chronic feeding of different diets containing vanaspati (TFA rich), palm oil (SFA rich) and sunflower oil (PUFA rich) at 10%level on 11β-HSD1 gene expression in rat retroperitoneal adipose tissue. 11β-HSD1 gene expression was significantly higher in TFA rich diet-fed rats compared to SFA rich diet-fed rats, which in turn was significantly higher than PUFA rich diet-fed rats. Similar trend was observed in the expression of CCAAT-enhancer binding protein-α (C/EBP-α), the main transcription factor required for the expression of 11β-HSD1. We propose that TFAs and SFAs increase local amplification of glucocorticoid action in adipose tissue by upregulating 11β-HSD1 by altering C/EBP-α-gene expression. The increased levels of glucocorticoids in adipose tissue may lead to development of obesity and insulin resistance, thereby increasing the risk of developing metabolic syndrome.

## Introduction

11β-hydroxysteroid dehydrogenase type 1 (11β-HSD1) plays an important role in the development of obesity and insulin resistance. 11β-HSD1 reactivates inactive glucocorticoids (11-dehydrocorticosterone in rodents, cortisone in humans) to their active forms (corticosterone in rodents, cortisol in humans) [1]. It is expressed in various tissues including liver and adipose tissue. Mice over-expressing 11β-HSD1 in adipose tissue develop visceral obesity and insulin resistance [2]. 11β-HSD1 knockout mice are resistant to diet- induced obesity and have exhibited improved insulin sensitivity [3]. CCAAT/enhancer -binding protein α (C/EBP α) directly regulates the expression of 11β-HSD1 [4], where as liver × receptor-α (LXR-α) down regulates it by indirect mechanisms [5].

Dietary fatty acids play an important role in cellular physiology by altering membrane fluidity, signal transduction and gene expression. They are also implicated in the development of metabolic syndrome (visceral obesity, insulin resistance, type 2 diabetes mellitus, dyslipidemia, hypertension, and increased cardiovascular risk profile). Trans fatty acids (TFAs) and saturated fatty acids (SFAs) are reported to play an important role in the development of obesity and insulin resistance [6-8]. The mechanisms through which TFAs and SFAs increase obesity and insulin resistance are poorly understood. TFAs and SFAs are shown to decrease the insulin sensitivity in rat adipose tissue by up regulating genes like resistin [9], on the contrary, polyunsaturated fatty acids like linoleic (18:2 n-6) acid are shown to improve insulin sensitivity and down regulate the lipogenic genes in adipose tissue [10]. Although previous studies reported the effect of high fat diet on 11β-HSD1 gene expression in adipose tissue of rodents and humans [11,12], presently no studies have reported the impact of dietary fat with varied fatty acid composition on the expression of adipose tissue11β-HSD1.

As 11β-HSD1 plays a key role in the adipose tissue metabolism and development of metabolic syndrome, a study was designed to assess the impact of dietary fats with high TFAs, SFAs and PUFAs contents on the expression of 11β-HSD1 in rat adipose tissue.

## Materials and methods

### Animals, diets and experimental design

Twenty four weanling female fischer rats were obtained from the National Centre for Laboratory Animal Sciences (NCLAS) and divided into three groups of 8 each and housed individually in a temperature (22 ± 2°C) and light controlled (12 h cycle) conditions. The study was approved by Institutional Animal Ethical Committee (IAEC). The animals were fed on isocaloric (1.78 kJ/100 g diet) semi-synthetic diet containing identical amounts of all dietary constituents, except the quality of dietary fats (fatty acid composition) for a period of 12 months. The salt and vitamin mixtures were prepared according to American Institute of Nutrition AIN-93[13]. The fatty acid compositions of various dietary oils (g/100 g) were estimated by Gas chromatography [14] and shown in Table 1. Vanaspati (Indian partially-hydrogenated vegetable oil) was used for the preparation of trans fatty acid rich diet (TFA rich diet), while palmolein and sunflower oils were used for the preparation of saturated fatty acid (SFA rich) and polyunsaturated fatty acid (PUFA rich diet) rich diets respectively. The animals had free access to water and food. At the end of the experiment, animals were sacrificed by CO_2 _inhalation and the retroperitoneal adipose tissue was dissected and frozen immediately in liquid nitrogen and stored at -80°C for RNA isolation.

**Table 1:** Fatty acid composition of dietary oils (g/100 g of oil).

Fatty acid	vanaspati	palmolein	sunflower
14:0	0.9	0.79	nd
16:0	38.4	35.6	6.55
16:1	nd	0.16	0.13
18:0	5.16	4.50	4.67
18:1t	20.1	nd	nd
18:1c	29.4	45.6	44.0
18:2	4.31	12.4	43.6
20:0	0.48	0.55	0.45
18:3	nd	0.25	0.44
∑ SFA	45.0	41.5	11.7
∑MUFA	29.4	45.6	44.0
LA	4.30	12.4	43.6
ALNA	nd	0.25	0.44

nd-not detected, ∑SFA-total saturated fatty acids, ∑MUFA-total monounsaturated fatty acids, LA-lenoleic acid, ALNA-alpha-linolenic acid.

Vegetable oils were purchased from a local market, and used as a source of dietary fat (10%).Vanapati, palmolien and sunflower oil were used for the preparation of trans fatty acid diet (TFA-diet), saturated fatty acid diet (SFA diet) and polyunsaturated fatty acid diet (PUFA- diet) respectively.

### Gene expression by semi quantitative reverse transcription-polymerase chain reaction

Total cellular RNA was extracted using a modification of the method of Chomczynski and Sacchi [15]. The integrity of the RNA was checked using 1% agarose gels stained with ethidium bromide. The RNA was quantified by spectrophotometric absorption at 260 nm. 1.0 μg of RNA was used to synthesize first strand cDNA. The reverse transcription (RT) reaction was carried out by incubating RNA with 0.5 μg oligo dT primer (Sigma) and 100 units of Molony murine leukemia virus reverse transcriptase (Finnzymes, Espoo, Finland) at 37°C for 60 min. Total reaction volume used in RT was 20 μL. An aliquot of cDNA was amplified in a 20 μL reaction mixture. PCR conditions were as follows, denaturation at 94°C for 1 minute, annealing for 45 seconds and polymerization for 70°C for I min with DyNAzyme II DNA polymerase (Finnzymes, Espoo, Finland). A final extension was carried out at 70°C for 7 min. The amount of RNA and the annealing temperature for different genes were standardized for linearity. Sequences of primers used for amplification are 11β-HSD1: forward primer-5'-GAGGAAGGGCTCCAG-3' and reverse primer-5'-GAGCAAACTTGCTTGCA-3'(NM_017080), LXRα: forward primer-5'-GCCCCATGGACACCTA-3' and reverse primer-5'-TGAGGGTCGGGTGCAA-3' (NM_031627), C/EBPα: forward primer-5'-GAGCCGAGATAAAGCCAA-3' and reverse primer 5'-CTTTCAGGCGACACCA-3' (NM_012524), β-actin: forward primer-5'- ACCAACTGGGACGACATGGA-3' and reverse primer-5'- TCTCAAACATGATCTGGGTCA-3' (NM_031144). β-actin gene was used as an internal control. After amplification, 8 μL of reaction mixture were electrophoresed on agarose gel (2%) in Tris-borate EDTA buffer (pH 8.2). The ethidium bromide stained bands were visualized by a UV-transilluminator and analyzed densitometrically using Quantity One software program (Bio-Rad, version 4.4.0). Each experiment was repeated three times for similar results.

### Statistics

Data were analyzed by one way ANOVA. Data are presented as mean ± SEM. Statistical significance was taken at level of P < 0.05 (two tailed).

## Results

### Effect of dietary fatty acid composition on bodyweight gain and insulin resistance

Body weight gain increased significantly by TFA and SFA rich diet-fed rats compared to PUFA rich diet-fed rats (Table 2). There is no significance difference in bodyweight gain between TFA and SFA rich diet-fed rats (Table 2). There were no significant differences in fasting plasma insulin and glucose levels among the three groups (data not shown). TFA and SFA rich diet-fed rats had significantly lower insulin stimulated glucose uptake in diaphragm compared to PUFA- rich diet fed rats and also there is no significance difference in insulin stimulated glucose uptake between TFA and SFA rich diet-fed rats (data not shown).

**Table 2:** Body weight changes in experimental groups

	PUFA-diet		SFA-diet		TFA-diet	
			
	Mean	SEM	Mean	SEM	Mean	SEM
Initial Body weight (g)	55.5	2.5	55	3.0	54	2.5
Final Body weight (g)	185	2.7^a^	220	3.5^b^	195	4.0^b^
Weight gain (g)	129	3.0^a^	165	4.5^b^	145	5.0^b^

Values are means ± SEM of 8 rats. Statistical significance was taken at P ≤ 0.05 level (by one-way ANOVA). Comparisons were made between the three experimental groups.

### Effect of dietary fatty acid composition on 11β-HSD1 gene expression

**Figure 1:**
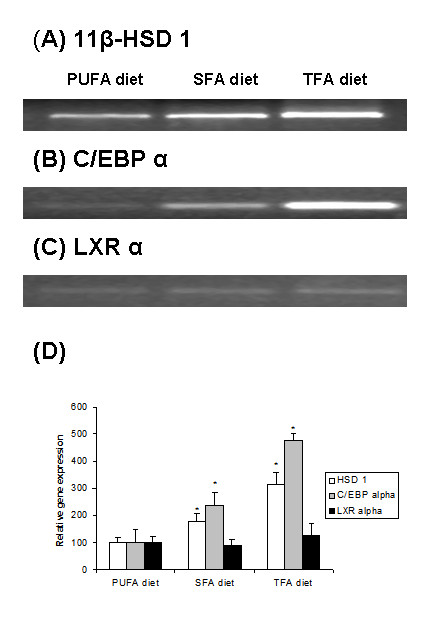
**Effect of various dietary fats on rat adipose tissue gene expression**. Rats were fed, one of the following isocaloric diets containing identical amounts of all dietary constituents except the type of dietary fat: trans fatty acid diet (TFA diet), saturated fatty acid diet (SFA diet), polyunsaturated fatty acid diet (PUFA diet). After 12 months of feeding, gene expression was analyzed in retroperitoneal adipose tissue by RT-PCR. (A) Representative photo of 11β-HSD1 PCR product, stained by ethidium bromide. (B) Representative photo of C/EBP-α PCR product, stained by ethidium bromide. (C) Representative photo of β-actin, PCR product (internal control), stained by ethidium bromide. (D) Relative 11β-HSD1, C/EBP-α and LXR-α mRNA expression was measured in relation to β-actin mRNA levels. Relative percentage gene expression of candidate gene in TFA- or SFA -fed rat RPWAT was calculated taking the relative gene expression observed in PUFA-fed rat RPWAT as 100. Values are mean ± SD, n = 3, (*, P < 0.05).

Our main aim was to determine whether 11β-HSD1 gene expression in rat adipose tissue was altered in response to chronic dietary feeding of TFA, SFA and PUFA rich diets. 11-βHSD1 mRNA levels increased significantly by 78.5*% *and 214% in RPWAT of SFA and TFA rich diet-fed rats respectively compared to those observed in PUFA-rich diet-fed rats (Fig. 1A&1D). Compared to SFA-rich diet, TFA-rich diet increased the 11-β HSD1 mRNA levels significantly by 76%, in RPWAT of rats (Fig. 1A&1D).

### Effect of dietary fatty acid composition on CCAAT/enhancer -binding protein α (C/EBP α) and Liver-X-Receptor-α (LXR-α) gene expression

C/EBP α mRNA levels increased significantly by 138% and 376% in RPWAT of SFA and TFA-rich diet -fed rats respectively, compared to those of PUFA rich diet-fed rats (Fig.1B&1D). TFA-diet increased the C/EBP α mRNA levels significantly by 172% in RPWAT as against those of SFA-diet fed rats (Fig.1B&1D). No differences in LXR-α mRNA levels among the groups were observed (Fig.1D).

## Discussion

The major finding of the present study is that, the dietary fatty acid composition regulates the 11β-HSD1 expression in rat adipose tissue. 11β-HSD1 was upregulated to a great extent in rat adipose tissue by TFA rich diet, compared to SFA rich diet, which in turn was higher than that observed in PUFA-rich diet (TFA > SFA> PUFA).

The role of 11β-HSD1 in the development of metabolic syndrome is confirmed by transgenic studies. Mice over expressing 11β-HSD1 in adipose tissue are modestly obese and develop all other features of metabolic syndrome including insulin resistance [2]. 11β-HSD1 knockout mice are resistant to diet induced obesity, showed improved insulin sensitivity [3]. In the present study, consumption of both SFA and TFA rich diets increased 11β-HSD1 gene expression in rat retroperitoneal adipose tissue, compared to PUFA-fed rats. In line with the increased 11β-HSD1 gene expression, both TFA- and SFA-diet fed rats showed significantly increased body weight gain and increased peripheral insulin resistance compared to PUFA rich diet-fed rats. Although there is significance difference in 11β-HSD1 gene expression levels of SFA and TFA rich diet-fed rats, this did not result in change in body weight gain or insulin resistance parameters of these two groups. This observation support the previous studies reporting the association between dietary trans and saturated fatty acids with development of obesity and insulin resistance [6-10] and also provides the new insight into the mechanism through which TFAs and SFAs increase the risk of obesity and insulin resistance.

The mechanism by which dietary fatty acids regulate the expression of 11β-HSD1 may be through Peroxisome proliferator-activated receptor-γ (PPAR γ). PPAR γ is known to down regulate the expression of 11β-HSD1 [16]. The regulation by these receptors is indirect and involves other yet unidentified transcription factors. PPAR γ has high affinity for unsaturated fatty acids compared to saturated fatty acids. Arachidonic (20:4 n-6) acid and its metabolites like 15-deoxy-Δ12, 14-prostaglandin J2 are high affinity ligands for PPAR γ [17]. In 3T3 L1 adipocytes, arachidonic acid has been shown to decrease the lipogenic gene expression through its conversion to prostaglandins [18]. As dietary lenoleic (18:2 n-6) acid, is the precursor for the synthesis of arachidonic (20:4 n-6) acid, the diet rich in this fatty acid (PUFA-rich diet) might have provided higher amounts of ligands for PPAR γ. This may be one of the possible mechanisms underlying the down regulation of 11β-HSD1 in adipose tissue of PUFA-fed rats. Both dietary SFAs and TFAs are shown to decrease the levels of n-6 PUFA (linoleic acid and arachidonic acid) in adipocyte plasma membrane phospholipids [18]. Decreased contents of linoleic and arachidonic acids might have decreased the availability of ligands for PPAR γ, which could have resulted in the upregulation of 11β-HSD1 in adipose tissue of TFA and SFA-diet fed rats.

Our data suggest that dietary fatty acids may regulate the 11β-HSD1 gene expression directly by altering C/EBP α gene expression. CCAAT/Enhancer-binding proteins are essential for the preadipocyte differentiation and maintenance of adipocyte phenotype [19]. C/EBP α is a known potent transcriptional activator of 11β-HSD1 [5]. It has already been shown that in adipocytes, C/EBP α regulates the expression of key genes involved glucose and lipid metabolism [20-22]. Chronic challenging with TFA and SFA rich diets increased the expression of C/EBP α-m RNA levels in rat adipose tissue compared to PUFA-rich diet. Between TFAs and SFAs, TFA appeared to be more potent stimulator of C/EBP α. The mechanisms by which dietary fatty acids regulate the C/EBP α are unclear.

In conclusion, diets rich in trans fatty acids and saturated fatty acids increased 11β-HSD1 gene expression in RPWAT of rats as compared PUFA enriched diet. The differential regulation of 11β-HSD1 gene in RPWAT of rat by different dietary fatty acids may be through regulation of C/EBP-α gene. These results suggest that in adipose tissue, diets rich in TFAs and SFAs increase the local amplification of glucocorticoids than PUFA-rich diets. Thus, the increased local conversion of inactive to active glucocorticoids in adipose tissue may increase the risk of developing obesity and insulin resistance.

## Competing interests

The authors declare that they have no competing interests.

## Authors' contributions and information

SSSVP proposed the hypothesis, carried out PCR analysis and wrote the manuscript. JSM collected the tissues and isolated the RNA. UKP and QSSYH designed and carried the experiment. VA drafted the manuscript and had overall supervision and gave final approval of the manuscript to be published. All authors have read and approved the final manuscript.
